# One-step nucleic acid amplification for intraoperative diagnosis of lymph node metastasis in lung cancer patients: a single-center prospective study

**DOI:** 10.1038/s41598-022-11064-4

**Published:** 2022-05-04

**Authors:** Kei Namba, Ken Suzawa, Kazuhiko Shien, Akihiro Miura, Yuta Takahashi, Shunsaku Miyauchi, Kota Araki, Kentaro Nakata, Shuta Tomida, Shin Tanaka, Kentaroh Miyoshi, Shinji Otani, Hiromasa Yamamoto, Mikio Okazaki, Seiichiro Sugimoto, Junichi Soh, Masaomi Yamane, Shinichi Toyooka

**Affiliations:** 1 Department of Pathology, Memorial Sloan Kettering Cancer Center, New York, NY 10065 USA; 2 Department of General Thoracic Surgery and Breast and Endocrinological Surgery, Okayama University Graduate School of Medicine, Dentistry, and Pharmaceutical Sciences, 2-5-1 Shikata-cho, Kita-ku, Okayama, 700-8558 Japan; 3 Center for Comprehensive Genomic Medicine, Okayama University Hospital, Okayama, 700-8558 Japan; 4 Division of Thoracic Surgery, Faculty of Medicine, Kindai University, Osaka-Sayama, 589-8511 Japan

**Keywords:** Cancer therapy, Lung cancer, Tumour biomarkers

## Abstract

One-step nucleic acid amplification (OSNA) is a rapid intraoperative molecular detection technique for sentinel node assessment via the quantitative measurement of target cytokeratin 19 (*CK19*) mRNA to determine the presence of metastasis. It has been validated in breast cancer but its application in lung cancer has not been adequately investigated. 214 LNs from 105 patients with 100 primary lung cancers, 2 occult primary lung tumors, and 3 metastatic lung tumors, who underwent surgical lung resection with LN dissection between February 2018 and January 2020, were assessed. Resected LNs were divided into two parts: one was snap-frozen for OSNA and the other underwent rapidly frozen histological examination. Intraoperatively collected LNs were evaluated by OSNA using loop-mediated isothermal amplification and compared with intraoperative pathological diagnosis as a control. Among 214 LNs, 14 were detected as positive by OSNA, and 11 were positive by both OSNA and intraoperative pathological diagnosis. The sensitivity and specificity of OSNA was 84.6% and 98.5%, respectively. The results of 5 of 214 LNs were discordant, and the remainder all matched (11 positive and 198 negative) with a concordance rate of 97.7%. Although the analysis of public mRNA expression data from cBioPortal showed that *CK19* expression varies greatly depending on the cancer type and histological subtype, the results of the five cases, except for primary lung cancer, were consistent. OSNA provides sufficient diagnostic accuracy and speed and can be applied to the intraoperative diagnosis of LN metastasis for non-small cell lung cancer.

## Introduction

Lung cancer is one of the most frequent cancers in humans and the leading cause of cancer deaths worldwide. In the clinical management of the disease, histological classification by staging is essential for an accurate therapeutic approach^1^.

The standard surgery for primary lung cancer is lobectomy and systematic lymph node (LN) dissection, including the hilar and mediastinal LNs^2,3^. However, in recent years, in some early-stage patients, limited lung resection and/or selective LN dissection have been frequently performed in clinical practice^4–6^. In such cases, rapid intraoperative diagnosis of the presence or absence of regional LN metastasis is performed, and the presence of metastases determines the optimal surgical procedure. Rapid intraoperative assessment of LN metastasis is usually performed by histopathological examination using frozen-section specimens, leading to more difficulties in the decision-making process than formalin-fixed specimens and requisition of a skilled pathologist. Thus, it can be undertaken at a limited number of medical institutes.

Currently, the molecular examination of cytokeratin 19 (CK19) expression is applied in clinical practice for the detection of LN metastasis. CK19 is a type I keratin with a low molecular weight that comprises the cytoskeleton of numerous epithelial cells of different organs but is not detected in any of the cellular components of LNs. It is highly expressed in epithelial tumors, and thus is an appropriate biomarker for detecting cancer metastasis. The one-step nucleic acid amplification (OSNA) assay (Sysmex Corp., Kobe, Japan) is a technique that calculates *CK19* mRNA expression in the entire LN tissue automatically. This technique utilizes a loop-mediated isothermal amplification (RT-LAMP) method to quantify *CK19* mRNA expression, enabling a fast, molecular, whole-node analysis^7–9^. Several studies comparing the OSNA assay with standard histopathological examination in patients with breast and colorectal carcinoma have been published, and the OSNA assay has been developed as an alternative intraoperative method for detecting tumor metastasis especially in breast cancer^7,10–16^. Additionally, there have been efforts to implement LN examination by OSNA for other malignancies such as endometrial^17,18^, gastric^19,20^, head and neck^21^, cervical^22,23^, thyroid^24,25^, and prostate cancer^26,27^. In terms of lung cancer, although several reports have been published on the potential of the OSNA assay, limited studies have directly demonstrated the correlation between OSNA and intraoperative pathological diagnosis^8^.

In this prospective study, we investigated the intraoperative diagnostic utility of OSNA to evaluate LN metastasis by comparing the results to those of conventional intraoperative frozen-section pathological diagnosis in lung cancer patients.

## Material and methods

### Patients

This prospective study was approved by the institutional review board of Okayama University Hospital, Okayama, Japan (approval number: 1802), and written informed consent was waived. Between February 2018 and January 2020, a total of 105 patients with operable primary lung cancer or metastatic lung tumors were examined. All surgical procedures were performed at the Department of Thoracic Surgery, Okayama University Hospital. The detailed characteristics of the 105 patients are provided in Table 1. In primary lung cancer patients, the pathological stage was based on the TNM classification 8th edition, and systematic nodal dissection was executed according to the well-proven scheme of the International Association for the Study of Lung Cancer (IASLC) of 2009^28^.

**Table 1:** Characteristics of the 105 cases in the study.

Characteristics	Patients n = 105 (%)
Age at diagnosis (range), y	70.3 (40–89)
**Sex, n (%)**	
Male	59 (56.2)
Female	46 (43.8)
**Histology, n (%)**	
Primary lung cancer	100 (95.2)
Ad	80
Sq	14
Other NSCLC	4
Small	2
Occult primary tumor	2 (1.9)
Metastatic lung tumor	3 (2.9)

NSCLC, non-small cell lung cancer; Ad, adenocarcinoma; Sq, squamous cell carcinoma.

Resected LNs for intraoperative pathological diagnosis were divided into two parts. One was subjected to rapidly frozen histological examination with HE (Hematoxylin–Eosin Stain) by pathologists, and the other was snap-frozen with liquid nitrogen and stored at − 80 °C until the OSNA assay. These sections were generated in the operating theater immediately after LN removal. The lung parenchymal tissue surrounding the LNs was macroscopically removed by the surgeon to avoid false-positive OSNA results due to contamination with normal lung tissue.

### OSNA assay

The OSNA assay uses *CK19* mRNA as a marker for the molecular diagnosis of LN metastasis, and the protocol for the OSNA assay was described previously^29–31^. In summary, one of the divided resected LNs was homogenized using Lynorhag lysis buffer (Sysmex Corp., Kobe, Japan). *CK19* mRNA in each lysate was amplified using LynoampBC gene amplification reagent (Sysmex Corp., Kobe, Japan). The results were determined using a "standard sample" diluted 10 times and a "diluted sample" diluted 100 times from the lysate. Then a 20-μl sample of each lysate was subjected to an RT-LAMP reaction. Amplification of *CK19* mRNA was detected by measuring the rise time on the basis of a standard curve using an RD-100i system (Sysmex Corp., Kobe, Japan). The results of the assay were expressed as *CK19* mRNA copy numbers per microliter. The cut-off for micrometastasis (+) of the standard sample was set at 250–5000 copies/μl; over 5000 copies/μl was considered as macrometastasis (+ +) and fewer than 250 copies/μl was considered negative^31^.

### Gene expression data from clinical tissue samples

As a source of clinical cancer tissue materials, we collected *CK19* mRNA expression data, which is available on cBioPortal (http://www.cbioportal.org/)^32,33^ and Gene Expression Omnibus (GEO)^34^. Obtained mRNA expression data was displayed on box plots where the box encloses the first to third quartiles, the bar inside the box represents the median, the whisker at the top indicates the maximum value excluding outliers, and the whisker at the bottom indicates the minimum value excluding outliers. Outliers were defined as values more than the third quartile + 1.5 × IQR or less than the first quartile − 1.5 × IQR, where IQR is the interquartile range.

### Institutional review board statement

The study was conducted according to the guidelines of the Declaration of Helsinki and approved by the institutional review board of Okayama University Hospital, Okayama, Japan (approval number: 1802).

### Informed consent statement

Informed consent was obtained from all subjects involved in the study.

## Results

### Concordance between OSNA assay and histological examination

In this prospective study, 214 LNs from 105 patients who underwent surgical lung resection with LN dissection were analyzed. There were 46 women and 59 men, and the median patient age was 70.3 years (range, 40–89 years). There were 100 primary lung cancer patients (80 adenocarcinomas, 14 squamous cell carcinomas, four other non-small lung cancers, and two small cell lung cancers) and five metastatic lung tumor patients (Table 1).

**Figure 1:**
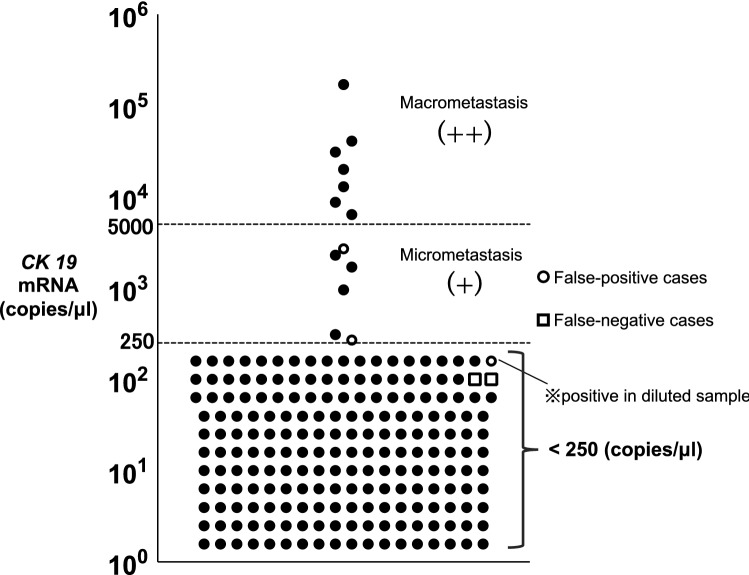
*CK19* mRNA copy numbers by OSNA assay (n = 214). The cut-off value for micrometastasis (+) of the standard sample was set at 250–5000 copies/μl; over 5000 copies/μl was diagnosed macrometastasis (+ +) and fewer than 250 copies/μl was diagnosed as negative^31^. Three open circles show false-positive cases and two open squares show false-negative cases.

Of the 214 LNs, 14 LNs were detected as positive with the OSNA assay. The copy number of *CK19* mRNA revealed by the OSNA assay is shown in Fig. 1. Seven LNs were detected as macrometastasis (over 5000 copies/μl), and six LNs were micrometastasis (250–5000 copies/μl). One LN was detected as positive only in the diluted sample; this is observed when the sample contains substances that have a negative effect on the PCR, so-called PCR inhibitors, causing a false-negative result. Dilution of a sample automatically results in the dilution of the PCR inhibitors and relieves the PCR inhibitory effect, meaning that the PCR reaction can be successful only in the diluted sample. In such a case, the sample is considered positive according to the manufacturer's instructions.

Eleven LNs were found to be positive by both OSNA assay and intraoperative pathological diagnosis, and 198 LNs were negative by both OSNA assay and intraoperative pathological diagnosis. Three LNs were positive according to the OSNA assay but negative by intraoperative pathological diagnosis, whereas two LNs were negative with the OSNA assay but positive by intraoperative pathological diagnosis. Our results showed a concordance rate of 97.7% with a kappa statistic of 0.802 (95% CI 58.6%–90.7%). The sensitivity of the OSNA assay compared with the intraoperative pathological diagnosis was 84.6% (95% CI 63.5%–94.8%), and specificity was 98.5% (95% CI 97.6%–99.7%). Positive predictive value and negative predictive value were 78.6% and 99.0%, respectively (Table 2). Next, the data was analyzed according to the number of patients, as shown in Table S1. The concordance rate was 96.2%, with a kappa statistic of 0.825 (95% CI 60.1%–89.6%). The sensitivity was 91.7% (95% CI 70.4%–98.4%) and specificity was 96.8% (95% CI 94.0%–97.6%). Positive predictive value and negative predictive value were 78.6% and 98.9%, respectively.

**Table 2:** Concordance between intraoperative pathological diagnosis and OSNA (number of lymph nodes).

Lymph nodes (n = 214)	Intraoperative pathological diagnosis	
	Positive	Negative
**OSNA assay**		
Positive (n)	11	3
Negative (n)	2	198
Sensitivity (%)	84.6	
Specificity (%)	98.5	
Positive predictive value (%)	78.6	
Negative predictive value (%)	99.0	
Concordance rate (%)	97.7	

### Discordant cases

We found a high correlation between the results obtained by OSNA assay and intraoperative pathological diagnosis. However, five LNs (four patients), less than 3% of all LNs analyzed, were discordant (Table 3).

**Table 3:** Cases with discordant results between intraoperative pathological diagnosis and OSNA assay.

Patient no.	Histological type	Lymph node station	IPD/OSNA*	*CK19* mRNA (copies/μl)
4	Sq	11 (interlobar node)	Negative/micro	2700
15	Ad	11s (interlobar node)	Negative/micro	< 250**
77	Ad	7 (subcarinal node)	Positive/Negative	< 250
		10L (hilar node)	Positive/negative	< 250
94	Ad	11 (interlobar node)	Negative/negative	270

Sq, squamous cell carcinoma; Ad, adenocarcinoma; IPD, interoperative pathological diagnosis.

*In the OSNA assay, macro, micro, and negative showed > 5000, 250–5000, and < 250 copies/uL of *CK19* mRNA, respectively.

**Micrometastasis detected in diluted sample.

Three LNs were diagnosed as negative for the intraoperative pathological diagnosis and positive for OSNA assay. All three LNs were detected as micrometastasis with OSNA. In contrast, all seven LNs detected as macrometastasis by OSNA assay were diagnosed as metastatic LNs by interoperative pathological diagnosis. In case #4, recurrence in the mediastinal LN occurred 6 months after surgery, requiring additional LN dissection. In case #15, the final pathological diagnosis showed metastasis in the #2R (upper paratracheal node) LN. In these cases, the OSNA method may have detected micrometastases that the intraoperative pathological diagnosis could not detect.

However, two LNs in the same patient case were negative by OSNA and diagnosed as metastasis by intraoperative pathological diagnosis. The final pathological diagnosis showed that two out of two #7 LNs and two out of four #10L LNs were positive.

### Primary lung cancers with minor histological subtype and metastatic lung tumors

In this study, there were six lung cancers with minor histological subtypes, including small cell lung cancer (SCLC), pleomorphic carcinoma (PC), adenosquamous cell carcinoma (AdSq), combined subtype with Sq and SCLC, and angiosarcoma. In addition, two occult primary tumor cases and three metastatic tumor cases were included (Table 4). A total of 17 LNs from 11 cases were analyzed, and the results from OSNA assay and intraoperative pathological diagnosis were consistent in all LNs, including three positive LNs. Previous reports reported low rates of *CK19* expression in PC or SCLC^35^; however, in this study, one LN in the PC case and one LN in a SCLC case were detected as macrometastasis by OSNA, consistent with intraoperative pathological diagnosis. In the one positive LN, which was from a metastatic case of renal cell carcinoma (RCC), *CK19* expression was detected as expected^28^. All four LNs from sarcoma cases were negative by both methods.

**Table 4:** Primary cases with minor histological subtype and metastasis cases.

Primary/meta	Patient no.	Primary organ	Histological type	Lymph node station	IPD/OSNA*	*CK19* mRNA (copies/μl)
Primary	3	Lung	Combined Sq and SCLC	5 (subaortic node)	Negative/negative	< 250
				11 (interlobar node)	Negative/negative	< 250
				12 (lobar node)	Negative/negative	< 250
	17	Lung	PC	9 (pulmonary ligament node)	Positive/macro	17,000
	25	Lung	SCLC	7 (subcarinal node)	Negative/negative	< 250
	52	Lung	SCLC	10R (hilar node)	Negative/negative	< 250
				11 s (interlobar node)	Negative/negative	< 250
	73	Lung	AdSq	4L (lower paratracheal node)	Negative/negative	< 250
				5 (subaortic node)	Negative/negative	< 250
				6 (para-aortic node)	Negative/negative	< 250
	78	Lung	Angiosarcoma	4R (lower paratracheal node)	Negative/negative	< 250
				10R (hilar node)	Negative/negative	< 250
Occult primary	2	Unknown	SCLC/large	4L (lower paratracheal node)	Negative/negative	< 250
	6	Unknown	SCLC	11i (interlobar node)	Positive/macro	6400
Meta	33	Kidney	RCC	6 (para-aortic node)	Positive/micro	2300
	43	Retroperitoneum	Sarcoma	10R (hilar node)	Negative/negative	< 250
	69	Bone	Sarcoma	11i (interlobar node)	Negative/negative	< 250

IPD, interoperative pathological diagnosis; Meta, metastasis; Sq, squamous cell carcinoma; SCLC, small cell lung cancer; PC, pleomorphic carcinoma; AdSq, adenosquamous cell carcinoma; RCC, renal cell carcinoma.

*In the OSNA assay, macro, micro, and negative showed > 5000, 250–5000, and < 250 copies/uL of *CK19* mRNA, respectively.

**Figure 2:**
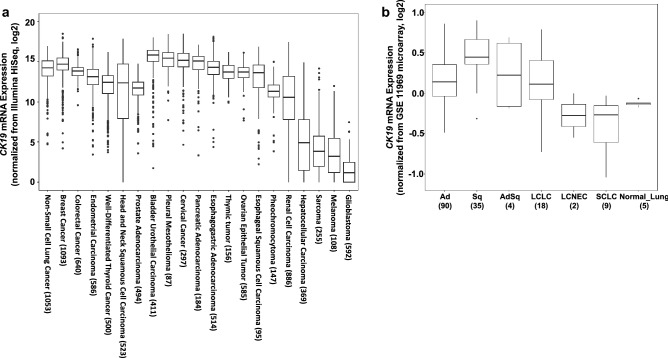
Boxplots of *CK19* mRNA expression data obtained from a public database. The box encloses the first to third quartiles, the bar inside the box represents the median, the whisker at the top indicates the maximum value excluding outliers, and the whisker at the bottom indicates the minimum value excluding outliers. (**a**) *CK19* mRNA expression data obtained from cBioPortal for patients with cancers of various types. The number of samples is shown in parentheses. (**b**) *CK19* mRNA expression microarray data obtained from GSE11969 for patients with lung cancers of various histological types. The number of samples is shown in parentheses.

To assess the utility of OSNA assay for lung cancers of minor histological subtypes and metastatic lung tumors further, *CK19* mRNA expression profiles for these cancer types were obtained from the public database (Fig. 2a). We queried 10,705 samples from 31 studies and plotted 9,575 samples in which mRNA expression levels were available. The expression of *CK19* in NSCLC showed a high level (log2 IQR; 13.2–15.1) similar to that of breast cancer (13.9–15.4), in which OSNA has been widely used in practical applications. Compared with other cancer types in which the OSNA method has been reported to be helpful for the detection of *CK19*, such as cervical (14.3–16.0), colorectal (13.3–14.3), endometrial (12.1–14.0), thyroid (11.0–13.3), head and neck (7.8–14.7), and prostate cancer (10.8–12.4), *CK19* expression levels in SCLC samples was equivalent. For renal cell carcinoma, which was detected as positive by OSNA in this study, the IQR of *CK19* expression was 7.8–13.2. However, sarcoma clearly expressed lower levels of *CK19* compared with other epithelial cancer types. For further assessment of the differences between the histological subtypes of lung cancer, *CK19* mRNA expression profiles of each histological subtype of clinical lung cancer tissue were obtained from GSE11969. According to the normalized microarray data shown in Fig. 2b, the expression of *CK19* in SCLC was significantly lower than that in NSCLC, but was not higher than that in normal lung tissue.

## Discussion

This prospective study demonstrated that the OSNA assay successfully detected LN metastasis in patients who underwent surgical lung resection with LN dissection, showing high specificity, high concordant rates, and negative predictive value. These results are consistent with several previous studies in NSCLC^8,9,36–38^. While these OSNA studies compared permanent histological diagnosis, the current study is the first prospective study that focuses on comparisons with intraoperative pathological diagnosis.

With regards to LNs with discordant results, three LNs were diagnosed as positive by the OSNA assay but negative by interoperative pathological diagnosis. In two of the three cases, mediastinal LN metastasis was identified in sites not examined by interoperative pathological diagnosis, or mediastinal LN recurrence was identified 6 months after surgery. There are three possibilities for these false-positive cases. First, OSNA might detect *CK19* with higher sensitivity because all three false-positive cases were diagnosed as micrometastasis with relatively low copy numbers, which intraoperative pathological diagnosis may not have detected. In fact, in two out of three false-positive cases, mediastinal LN metastasis was found within 6 months. Second, there is the possibility of allocation bias because the slice used for intraoperative pathological diagnosis might not contain metastatic cancer cells. In contrast, the OSNA assay makes it possible to analyze the whole LN, and thus can avoid an allocation bias that is attributed to the uneven localization of metastatic cancer cells. Third, contamination by surrounding normal lung tissues is a factor. It is known that *CK19* is expressed in normal epithelial tissue including normal lung tissue, but not in normal LN tissue, which may cause false-positive results in hilar LNs when the normal lung tissue is contaminated during hilar LN sampling^8^. Normal lung tissue needs to be completely removed from LN tissue to prevent the possibility of false-positive data.

In contrast, two LNs from one patient were diagnosed as negative by the OSNA assay but positive by interoperative pathological diagnosis. Allocation bias was suspected as the reason for this false-negative result, but it is challenging to identify the cause. Although the cause of the negative results cannot be determined in this study, it is possible that the LN sample used for OSNA did not contain metastatic cancer cells.

The overall results, with a sensitivity of 84.6% and a concordance rate of 97.7%, were equivalent to those reported in previous studies, with concordance rates, sensitivity, and specificity of 88.3%–96.2%, 79.7%–100%, and 87.6%–96.1%, respectively^9,18,36–38^.

With the increase in limited lung resection, such as segmentectomy or wedge resection, and selective LN dissection, the demand for rapid and precise intraoperative diagnosis of regional LN metastasis is expanding. In daily clinical practice, many intraoperative pathological examinations are performed in specialized hospitals and institutions. Pathologists are inevitably under intense pressure to conduct these intraoperative diagnoses quickly and accurately. In institutions without pathologists, this problem may be even more serious. Application of the OSNA assay in clinical practice may ease the burden on pathologists because most processes are automatic, and the results are quantifiable. A skilled technician can perform all steps of the OSNA assay process, from system setup to determining the status of LNs. Chen and colleagues reported that the mean turnaround time of the OSNA assay was less than 40 min, similar to other institutions^39^. Furthermore, there may be no differences in the diagnostic accuracy of LN metastasis using the OSNA assay among institutions worldwide. These situations provide more personalized surgical care to patients regardless of the number or availability of pathologists at each institution. In addition, because the whole specimen can be examined, OSNA has the advantage of low allocation bias and high sensitivity. Therefore, the OSNA assay can provide an alternative option for intraoperative pathological assessment of LN metastatic status.

We analyzed 11 cases other than primary lung Ad and Sq, and a 100% concordance rate was obtained in 17 lymph nodes. However, Nakagawa et al. highlighted that the OSNA assay should not be used for several histological types including PC and SCLC because of concerns about low *CK19* expression levels, even though the number of such samples was very small in their study^36^. In the current study, the OSNA assay was able to detect all three cases of LN metastasis in PC and SCLC cases, and there were no discordant LNs in other rare histological subtype cases and metastatic cases. Analysis using data obtained from a public database suggested that *CK19* mRNA expression in sarcoma and SCLC is lower than that in NSCLC and other cancer types for which OSNA is reported to be useful for *CK19* detection. In the current situation, for those cancer types with relatively low *CK19* expression such as sarcoma and SCLC, the data remains insufficient to determine the usefulness of the OSNA assay, and further studies are required for its clinical application.

## Conclusions

This prospective study demonstrated a high concordance between the OSNA assay and intraoperative pathological diagnosis. OSNA provides sufficient diagnostic accuracy and rapidity and can be applied to the intraoperative diagnosis of LN metastasis in lung cancer patients.

## Supplementary Information

Supplementary Information.

## Data Availability

The data presented in this study are available on request from the corresponding author.
